# AXL, an Important Host Factor for DENV and ZIKV Replication

**DOI:** 10.3389/fcimb.2021.575346

**Published:** 2021-03-22

**Authors:** Shengda Xie, Huiru Zhang, Zhenjie Liang, Xingmiao Yang, Ruibing Cao

**Affiliations:** Ministry of Education (MOE) Joint International Research Laboratory of Animal Health and Food Safety, College of Veterinary Medicine, Nanjing Agricultural University, Nanjing, China

**Keywords:** ZIKV, DENV, AXL, phosphatidylserine (PS), interaction, invasion

## Abstract

Flaviviruses, as critically important pathogens, are still major public health problems all over the world. For instance, the evolution of ZIKV led to large-scale outbreaks in the Yap island in 2007. DENV was considered by the World Health Organization (WHO) as one of the 10 threats to global health in 2019. Enveloped viruses hijack a variety of host factors to complete its replication cycle. Phosphatidylserine (PS) receptor, AXL, is considered to be a candidate receptor for flavivirus invasion. In this review, we discuss the molecular structure of ZIKV and DENV, and how they interact with AXL to successfully invade host cells. A more comprehensive understanding of the molecular mechanisms of flavivirus-AXL interaction will provide crucial insights into the virus infection process and the development of anti-flavivirus therapeutics.

## Introduction

The Flaviviridae family includes three viral genera, namely Flavivirus, Pestivirus, and Hepatitis C virus, with a total of more than 70 viruses. Zika virus (ZIKV) and dengue virus (DENV) which are critically important pathogens belong to the Flavivirus genus.

DENV, the most prevalent arbovirus in the world, is widely popular in tropical and subtropical countries (Uno and Ross, 2018). It has four different antigenic serotypes (DENV1–4), which can cause dengue fever after the bite of an infected mosquito, with a global estimate of around 3.6 billion people and 40% of the world at risk for infection each year (Bhatt et al., 2013). With the process of urbanization, human migration, climate change, and damage to vegetation, the cases of DENV have increased by 30-fold (Chretien et al., 2015). Although Dengue is not a newly emerging disease, its clinical manifestations are constantly changing in recent years (Calderon-Pelaez et al., 2019). DENV can cause a wide spectrum of clinical manifestations, from asymptomatic and mild fever to fatal DENV shock syndrome (Diamond and Pierson, 2015). Although a high probability of manifesting itself as a self-limiting illness in the first infection, the antibody-dependent enhancement (ADE) effect increases the morbidity and mortality in the second infection with different serological viruses (Frei et al., 2018). Due to the lack of specific therapeutic drugs, the development of a vaccine against DENV is imminent.

ZIKV, an ancient virus, was originally discovered in the blood of a rhesus macaque in Uganda in 1947 and was subsequently isolated from *Aedes* mosquitoes (Ming et al., 2016). It was not until 2007 that ZIKV caused an outbreak in the Pacific Ocean and began to spread throughout the Latin Americas in 2015, did we realize its harm to public health and regard it as an international public health emergency (PHEIC) (Musso et al., 2019). Unlike other flaviviruses, ZIKV which is the only virus known to be teratogenic can cause fetal infection through vertical transmission, leading to congenital ZIKV syndrome (Ngono and Shresta, 2018; Weaver et al., 2018). Besides, ZIKV can cause severe Guillain-Barre syndrome (a post-infectious autoimmune poly-Neuropathy, GBS) and testicular atrophy in adult males (Uraki et al., 2017; Ngono and Shresta, 2018). The presence of virus particles in the testes (Sertoli cells, Leydig cells, and spermatogonia), semen, and sperm may have the potential for sexual transmission (Deng et al., 2020).

TAM proteins, including Tyro3, AXL, and Mer, are cell surface receptor tyrosine kinases (RTKs) (Graham et al., 2014). In 1991, Lai identified 13 novel genes including TAM based on the homology of RTK and classified AXL, Tyro3, and Mer as a unique subgroup (Lai and Lemke, 1991). TAM proteins need to bind with ligands to exert physiological functions. Currently, growth-arrest-specific 6 (Gas6) is known to bind and activate three receptors, while proteins S (Pros1) is a ligand only for Tyro3 and Mer (Stitt et al., 1995; Mark et al., 1996). The only ligand, Gas6, binds to AXL and transmits various signals from the extracellular matrix to the cytoplasm to regulate many physiological processes. These physiological activities include clearance of apoptotic cells (Ravichandran, 2010; Fourgeaud et al., 2016), regulation of the innate immune response (Rothlin et al., 2007; Trahtemberg and Mevorach, 2017), drug resistance and metastasis of many cancers (Zhang et al., 2012; Akalu et al., 2017), and prominently, the infection of enveloped viruses (Shimojima et al., 2007; Meertens et al., 2012; Savidis et al., 2016). Thus, flavivirus, as a type of enveloped virus, how to invade cells through AXL is the major topic of discussion in this review.

## Molecular Biology of DENV and ZIKV

**Figure 1:**
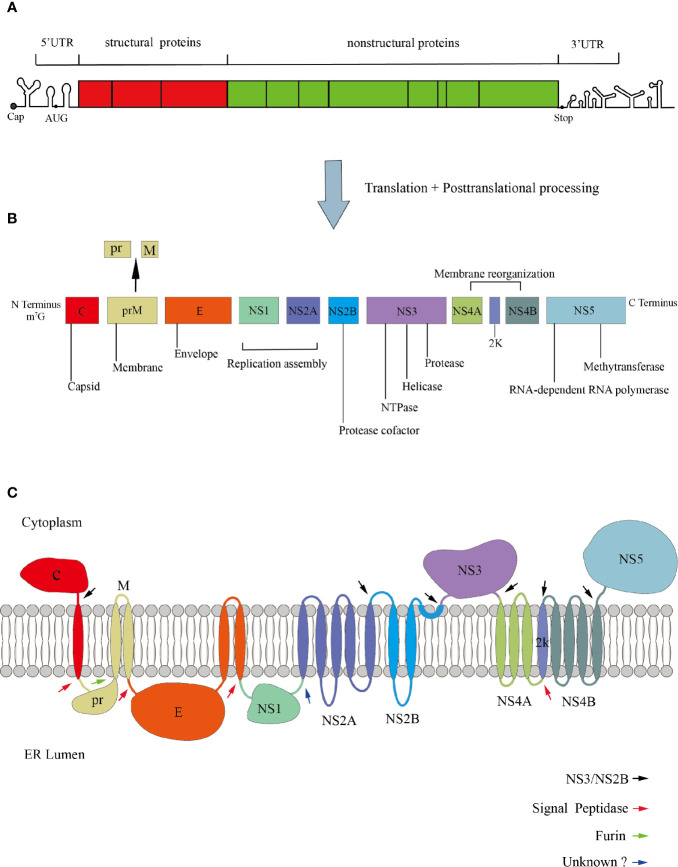
Genomic organization of Flavivirus. **(A)** The genome is divided into UTR and ORF. **(B)** The ORF encodes three structural proteins, seven non-structural proteins, and a signal peptide 2K with 17 amino acids. **(C)** Polyprotein membrane topology. The black arrows indicate the cleavage site of the viral protease NS3/NS2B. The red arrows denote the cleavage site of the host signal peptidase. The green arrow indicates the cleavage of prM to M and pr. The blue arrow denotes the cleavage site of an unknown host protease.

ZIKV and DENV are enveloped RNA viruses, with positive-sense and single-stranded RNA genome of ~11kb in length. The genome contains a methylated cap at its 5’ end but no polyA tail at its 3’ end (Barrows et al., 2018). The viral genome consists of three parts: the 5’ and 3’ non-translated regions (UTR), and the translated region (also known as open reading frame, ORF) (Rice et al., 1985; Lindenbach and Rice, 2003). The two structures in the 3’UTR that can inhibit the 5’ to 3’exonuclease Xrn1 are important for the formation of the subgenomic flaviviral RNA (sfRNA). Because of their ability for exonuclease-resistant, they play an important role in disrupting the innate immunity responses of hosts and promoting viral replication (Chapman et al., 2014; Akiyama et al., 2016; Zhang et al., 2020). The ORF is translated into a polyprotein, which is proteolytically processed by both host and virus proteins to form the final mature virus particles. The translated products contain three structural proteins (capsid, C; premembrane, prM; and envelope, E) and seven non-structural proteins (NS1, NS2A, NS2B, NS3, NS4A, NS4B, and NS5) (Lindenbach and Rice, 2003) (**Figure 1**). The cleavage of C and prM proteins is mainly caused by the viral protease NS3 and the cell signal peptidase from the cytoplasm and the endoplasmic reticulum(ER) lumen, subsequently, releasing the mature C protein into the cytoplasm, while another small molecule immature C protein is left in the ER lumen (Lobigs, 1993; Amberg and Rice, 1999). prM-E and E-NS1 are only cleaved by cell signal peptidase (Markoff, 1989; Barrows et al., 2018; Yun and Lee, 2018). However, the prM is just a precursor protein, which forms spike-like non-infectious particles with C and E proteins in ER (Perera and Kuhn, 2008). Under low pH conditions, prM will be cleaved into M protein by furin protease in the trans-Golgi network and form mature virus particles (Mukhopadhyay et al., 2005). Once cleaved, the pr protein will still bind to the E-dimer, preventing premature viral membrane fusion with the host cell until the infectious viral particles are released (Yu et al., 2008).

The structure of virus particles plays an important role in the process of invading host. The viral entry into target cells depends on PS contacts with its cognate receptors, such as TIM and TAM (Morizono et al., 2011; Meertens et al., 2012; Niu et al., 2018), so it is necessary to understand the structural proteins of viral particles. Mature DENV virus particles are icosahedral symmetrical spherical bodies with a diameter of about 50 nm. The surface of the virus is a glycoprotein coat made of 180 copies of E protein and M protein (Kuhn et al., 2002). It is generally considered that E protein is divided into three regions: E-DI required for the rearrangement of E protein structure, E-DII involved in the pH-mediated fusion with the host cell membrane, and ED-III contains the receptor-binding region. Besides, DI bridges DII and DIII (Rey et al., 1995). The structure of ZIKV is roughly similar to other flaviviruses. But some subtle differences in structural proteins may explain the different cellular tropism and pathogenicity of ZIKV. In fact, DENV has two glycosylation sites at Asn67 and Asn154, while ZIKV has only one glycosylation site at Asn154 within the glycoprotein (Mondotte et al., 2007; Zhang et al., 2013a). The loss of N^154^-glycosylation modifications can reduce the transmission capacity of flaviviruses in mouse and mosquito models (Fontes-Garfias et al., 2017; Wen et al., 2018).

Phosphatidylserine is the most abundant negatively charged phospholipid in eukaryotic membranes. In healthy cells, almost 100% of PS is confined inside the bilayer, facing the cytoplasmic leaflets. When cells are apoptotic, macrophages recognize PS exposed on the cell surface and clear apoptotic cells (Nagata, 2018; Lemke, 2019). Flavivirus particles budding from ER which is the source of PS in its envelope. The PS exposed on the surface of the viral envelope can mask the virus as apoptotic bodies, tricking the cells into engulfing virus particles (Moller-Tank and Maury, 2014). Although the virus can expose PS during infection, it is still unknown how the membrane flips inside out and the PS can be expressed on the surface of virion envelope (Maginnis, 2018).

## Biology of AXL

**Figure 2:**
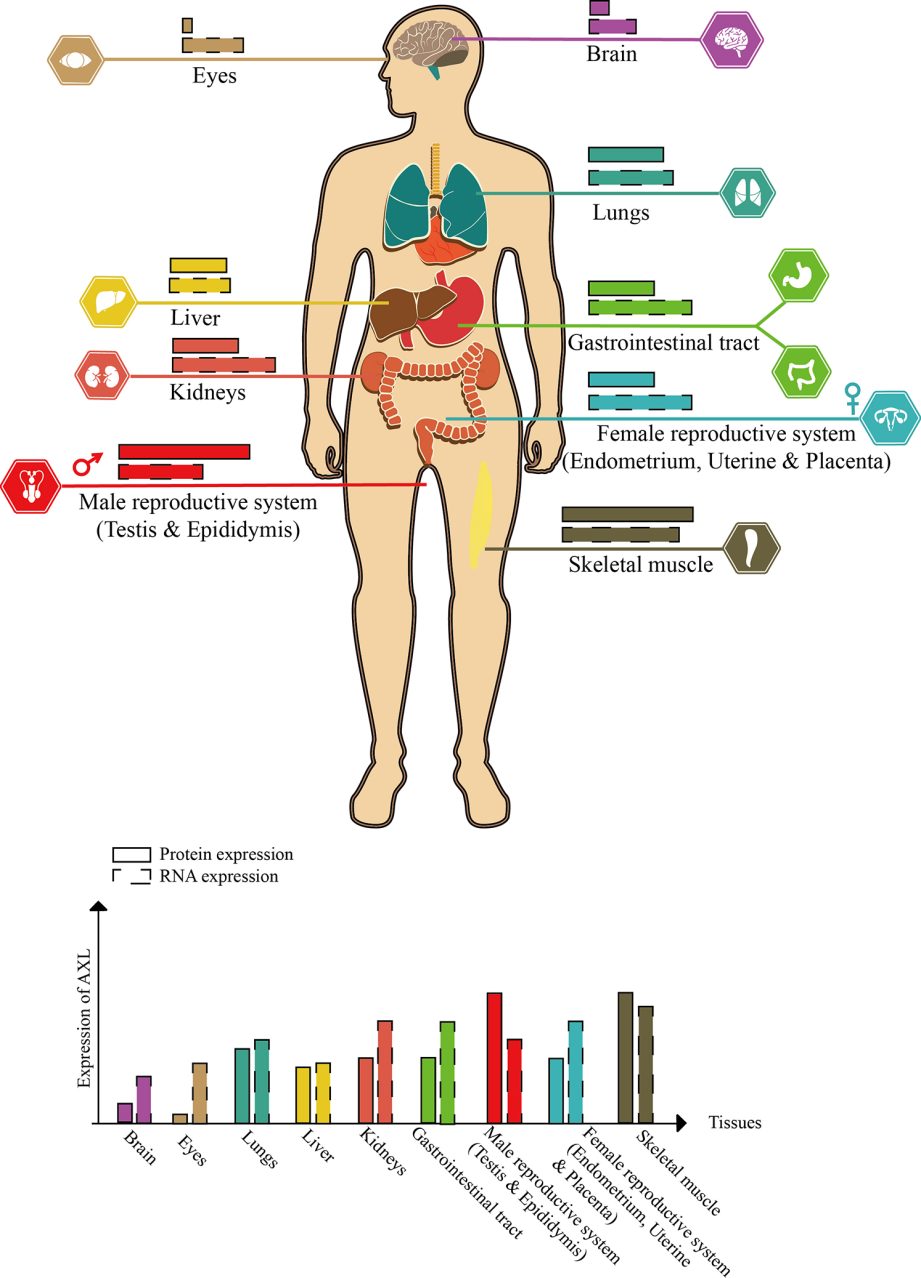
Schematic diagram of AXL expression in human tissues (this figure is adapted from the data of “THE HUMAN PROTEIN ATLAS” website. For specific data, please refer to https://www.proteinatlas.org/ENSG00000167601-AXL/tissue).

AXL, also known as UFO, was discovered from two patients with chronic myeloid leukemia in 1988 (O’Bryan et al., 1991). The human *AXL* gene which encodes 20 exons is ubiquitously expressed in various human organs, including brain, heart, liver, kidney, testis, uterus, and skeletal muscle (Shen et al., 2020) (**Figure 2**).

## Structure of AXL

AXL is a transmembrane receptor with a size of 100∼140 kd, which contains an extracellular (N-terminal) domain and an intracellular(C-terminal) tyrosine kinase domain (Korshunov, 2012). The intracellular domain is a tyrosine kinase domain with autophosphorylation properties. The extracellular domain contains two immunoglobulin-like (Ig) repeats and two fibronectin (FN) type III motifs, which have the characteristics of adhesion molecules and tyrosine kinase activity and can bind to its ligand (Gas6) (Myers et al., 2019). The characteristics of Ig-like and FN type III extracellular domains classify AXL (along with Tyro3 and Mer) as TAM family of RTKs.

**Figure 3:**
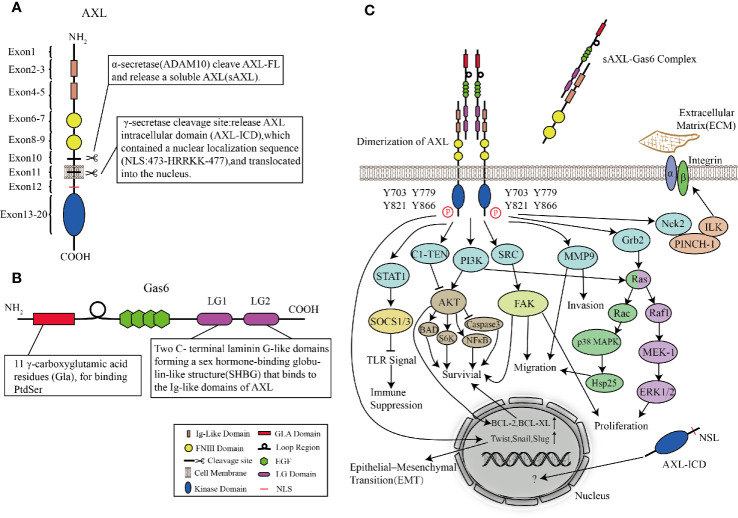
Structures of AXL and Gas6, and AXL signaling pathways. **(A, B)** Molecular structures of AXL **(A)** and Gas6 **(B)**. **(C)** AXL binds to the ligand Gas6, and then the tyrosine kinase domain is activated, mediating cascade reactions such as cell survival, proliferation, migration, invasion, immune suppression, and cytoskeleton dynamics.

The signal peptide of AXL is located within exon1, which can guide the transfer of newly synthesized AXL to the cell membrane. The exons 2–5 of AXL form two Ig-like domains, which are used to bind the sex hormone-binding globulin (SHBG) region of Gas6. The affinity between Gas6 and AXL is 3–10 times higher than Mer and Tyro3 (Shen et al., 2020). In the AXL-Gas6 complex, both of the Ig-like domains of AXL are simultaneously connected to the first laminin G-like domain in Gas6 (Sasaki et al., 2006). This special and ingenious combination prevents the incorrect combination of Gas6-Gas6 or AXL-AXL. The FNIII domains located in exons 6–9 provide the basis for AXL adhesion. It has been shown that the transmembrane domain near exon10 and exon11 can be cleaved by proteases to produce soluble AXL fragments (Weinger et al., 2009). Interestingly, in human non-small cell lung cancer cells (HCC827), α-secretase (ADAM10) can cleave the full-length AXL (AXL-FL) and release the extracellular domain into the blood termed soluble Axl (sAxl). These soluble extracellular domains shed from the full-length AXL still retain the ability to bind to Gas6. The structure composed of transmembrane domain and intracellular domain (AXL-CTF) can also be cleaved by γ-secretase. After being cleaved, AXL intracellular domain (AXL-ICD), which contains a nuclear localization sequence (NLS) located at exon12, is released from the plasma membrane and transferred to the cytoplasm and nucleus (Lu et al., 2017). Exons13–20 are intracellular domains with catalytic protein tyrosine kinases (**Figure 3A**).

## AXL Ligand: Gas6

Vitamin K-dependent protein Gas6, the only ligand known for AXL, was first discovered in embryonic mouse NIH 3T3 cells (van der Meer et al., 2014). Under conditions of serum-starved NIH 3T3 cells, Gas6, which shares approximately 43% of the amino acid sequence with protein S, is one of the up-regulated “growth arrest-specific” genes (Schneider et al., 1988; Law et al., 2018). Unlike the thrombin-sensitive cleavage site between the Gla and EGF-like domains of protein S, the region in Gas6 is not susceptible to be cleaved by the serine proteases (van der Meer et al., 2014). The N-terminus is Gla domain, a protein containing γ-carboxylated glutamic acid residues. In a vitamin K-dependent reaction, its glutamic acid residues are carboxylated at the free γ-hydroxyl position which greatly increases the ability to bind Ca^2+^. Therefore, Gla can undergo the calcium-dependent structural transformation, and thus can bind to phosphatidylserine (PS) residues with high affinity (Tanabe et al., 1997; Rajotte et al., 2008; Tsou et al., 2014). Following the Gla domain are a loop region (a disulfide bridge) and four epidermal growth factor (EGF)-like domains. EGF-like repeats consist of β-sheets containing six conserved cysteine residues to form three intra-domain disulfide bonds (Rothlin et al., 2015). Besides, each EGF-like domain contains a consensus sequence for the β-hydroxylation of Asp and Asn residues, whose existence of this structure is related to the high affinity of Ca^2+^ (Wu et al., 2017). The C-terminal of Gas6 is a sex hormone-binding globulin-like structure (SHBG) composed of two laminin G (LG)-like domains. LG is a matrix protein that can interact with receptors on the cell surface (Manfioletti et al., 1993; Wu et al., 2017) (**Figure 3B**). By observing the crystal structure of the minimal AXL-Gas6 complex, the lg1 and lg2 domains of AXL are connected in an antiparallel arrangement of the edge β strands to form a continuous β-sheet cross-molecular junction.

Other experiments show that the combination of AXL and Gas6 is divided into two steps. First, LG1 and the two lg-like domains are combined with high affinity, and then lateral diffusion of such 1:1 complexes leading to the formation of a dimeric signaling complex. At the main binding site, both AXL and Gas6 contain several charged residues forming part of the polar β-sheet surface. However, Pros1 does not have these charged residues, which may be the reason for its inability to bind to AXL (Hafizi and Dahlback, 2006; Sasaki et al., 2006; Linger et al., 2008).

In addition to the common AXL/Gas6 activation pathway, AXL can also form Gas6-independent heterodimers with other molecules (TYRO3, Mer, and epidermal growth factor receptor) to initiate intracellular signaling when AXL is overexpressed or under oxidative stress (Myers et al., 2016; Ray et al., 2017). These atypical activation pathways also illustrate the complexity of AXL activation.

## AXL Signaling Pathways

After AXL is activated, the intracellular tyrosine residues are autophosphorylated and dephosphorylated, and effector molecules or adaptor proteins containing SH2, PTB, or other phosphotyrosine-binding domains are recruited to these phosphotyrosine residues (Gay et al., 2017). Four tyrosine residues (Y703, Y779, Y821, and Y866) are considered as phosphorylation sites. These residues are involved in binding AXL with subunits of phosphatidylinositol 3-kinase (PI3K), growth factor receptor-bound protein 2 (Grb2), and Src tyrosine kinase (SRC) (Wium et al., 2018; Zhu et al., 2019). In a cell type-dependent context, AXL-activated downstream cascade signaling pathways such as the Grb2/Ras/Raf/MEK/ERK, PI3K/Akt, and SRC signaling pathways can mediate survival, proliferation, migration, invasion, immune suppression, and cytoskeleton dynamics. Gas6/AXL signaling promotes the growth and survival of multiple cell types by activating the MAPK/ERK and PI3K signaling pathways (Antony and Huang, 2017). The MAPK/ERK cascade is usually involved in proliferation, while PI3K signaling pathway is involved in cell survival. In leukemia cell lines, early experiments showed that AXL mediated cell proliferation *via* activation of Grb2, Ras, Raf1, MEK-1, and ERK1/2 pathways (Fridell et al., 1996). Stimulation of mitogen-activated protein (MAP) kinase (p38) by AXL is partly due to its ability to bind to the adaptor protein Grb2. Studies on GnRH neurons showed that AXL guided these cells to migrate from olfactory plaques to the forebrain *via* PI3K, Ras, Rac, p38 MAPK, and Hsp25 signaling pathways, leading to actin reorganization (Allen et al., 2002; Nielsen-Preiss et al., 2007). Large numbers of experiments proved that the activation of AXL and PI3K/Akt was related to multiple downstream cascade reactions converging on protecting cells from apoptosis. Akt not only activates ribosomal protein S6 kinase (S6K) but also phosphorylates a pro-apoptotic protein, BCL2-associated agonist of cell death (Bad) (Goruppi et al., 1997; Goruppi et al., 1999; Lee et al., 2002). In addition, Gas6/AXL signaling also increases the expression of antiapoptotic proteins, mediates phosphorylation of NFκB, and inhibits proapoptotic proteins such as caspase 3 (Goruppi et al., 1999; Demarchi et al., 2001; Hasanbasic et al., 2004). Both of AXL and Gas6 involved in cell survival are indispensable. AXL without Gas6 cannot be activated and induce downstream cascade reactions, whereas, Gas6 stimulated *AXL^−/−^* mice fibroblasts did not increase cell survival rate (Bellosta et al., 1997). However, C1-TEN is considered as a negative regulator of AXL-mediated PI3K/AKT signaling to reduce cell survival (Hafizi et al., 2005).

In addition, Nck2 is involved in linking AXL with other signaling complexes. The interaction of AXL and Nck2 can connect AXL to a ternary complex consisting of the particularly interesting new cysteine-histidine-rich protein (PINCH) and integrin-linked kinase (ILK), which is a major component of signaling platforms at focal adhesions, thereby enabling AXL to regulate cytoskeleton dynamics (McCarty, 1998; Lal et al., 2009; Verma et al., 2011). In inflammatory breast cancer cell lines, depletion of AXL-stabilizing protein TIG1 reduces the expression of Matrix metalloproteinase 9 (MMP9), which has been identified as an essential regulator for the AXL-mediated invasion (Tai et al., 2008; Koorstra et al., 2009; Han et al., 2013; Wang et al., 2013). Similarly, Src-family kinase activity is related to Gas6-mediated proliferation, survival, and neuronal migration (Goruppi et al., 1997; Nielsen-Preiss et al., 2007; Laurance et al., 2014). In human breast cancer epithelial cells, *SLUG* and *SNAIL* increase the expression of AXL, which indicates that AXL, as an important factor of the epithelial-mesenchymal transition (EMT) process, is involved in metastasis in cancer cells (Gjerdrum et al., 2010; Feneyrolles et al., 2014; Kong et al., 2020) (**Figure 3C**).

Different with its roles in proliferation, migration, and cell survival, studies of macrophages and dendritic cells (DCs) showed that AXL was pleiotropic inhibitor of the innate immune response to pathogens (Rothlin et al., 2007). Because of outbreaks of ZIKV and DENV, the roles of AXL in the process of flavivirus infections have become more and more clear.

## PS Receptor AXL Interacts With ZIKV and DENV

**Figure 4:**
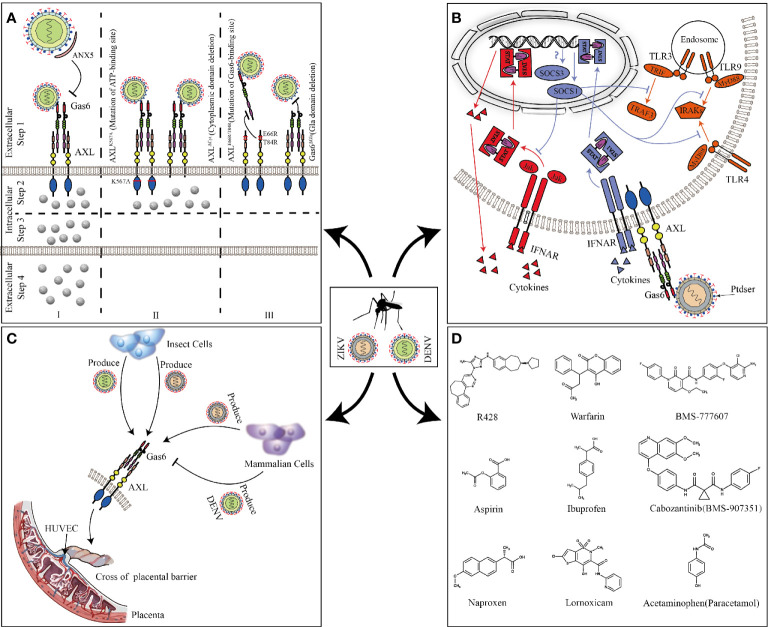
AXL promotes ZIKV and DENV replication. **(A)** Full-length AXL promote DENV replication. ANX5 competitively binds PS, blocking the binding of DENV to Gas6 (zone I). AXL^K567A^ and AXL^ΔCyt^ promote the binding and internalization of DENV (Zone II step2) but are unable to enhance DV infection (Zone II step3 and 4). Mutation of the Gas6 binding site in AXL can effectively inhibit DENV-Gas6 complex binding to AXL (zone III). **(B)** The feedback inhibition of the innate immune response to ZIKV. ZIKV-Gas6 complex activates AXL and up-regulates SOCS1 expression in a STAT1/STAT2-dependent manner (blue) to inhibit TLR signaling pathways and cytokine signaling (red). **(C)** ZIKV and DENV produced by insect cells or mammalian cells cross the placental barrier to infect HUVECs through AXL. **(D)** The chemical structure of the AXL inhibitors that have been reported to block ZIKV and DENV infections.

Most research about AXL focused on cancer and cell survival. It was not until 2007 that Rothlin discovered AXL and other TAM families could be used as inhibitors of innate immune responses, which opened the beginning of future research on AXL in flavivirus (Rothlin et al., 2007). In 2012, Meertens discovered AXL and other PS receptors can mediate DENV entry (Meertens et al., 2012) (**Figure 4A**). DENV used Gas6 to bridge AXL and PS, thereby promoting its replication (Zone I). The presence of the ligand Gas6 was indispensable for virus attachment. Pretreatment of DENV with ANX5 (a PS-binding protein) or mutation of the Gas6 binding site in AXL can effectively inhibit viral infection (Zone I and III). Interestingly, the deletion of the AXL intracellular domain or mutation of the ATP binding site did not change the internalization of DENV (Zone II step2) but affected the late replication of the virus to produce the progeny virus (Zone II step3, 4). This indicated that the intracellular kinase region of AXL was essential for infection of DENV but dispensable for viral entry. However, this article failed to elaborate on its deeper mechanism. Subsequently, by constructing a pseudotyped virus, Bhattacharyya discovered that enveloped viruses suppressed innate immune responses by activating TAM receptors, which may explain that why AXL intracellular domain deletion or ATP binding site mutant cannot promote DENV replication (Bhattacharyya et al., 2013).

Compared with DENV, ZIKV seems to have a higher affinity with AXL. A large number of experiments *in vitro* have proved that AXL is a candidate receptor for ZIKV. When sucking blood, *Aedes* mosquito deposits ZIKV in the epidermis and dermis of the bitten host. As the first barrier of innate immunity, multiple cells (including immature dendritic cells and epidermal keratinocytes) are permissive to ZIKV infection, and these cells highly expresses AXL (Hamel et al., 2015; Laureti et al., 2018). Besides the skin, AXL is expressed in various organs such as the brain, eyes, and reproductive organs (see **Figure 2** for details). ZIKV has shown strong neurotropism especially neural progenitor cells (NPCs), causing extensive neuropathy. Samples from mid-neurogenesis have shown that ZIKV has a high infection rate in ventricular zone (VZ) and subventricular zone (SVZ) (Retallack et al., 2016; Meertens et al., 2017). Interestingly, at mid-neurogenesis, AXL is expressed in a highly reproducible pattern throughout the cortex, with strong expression bordering the lateral ventricle and in the outer subventricular zone, which is consistent with ZIKV infection tropism zone (Nowakowski et al., 2016). Furthermore, AXL is overexpressed in multiple developing human brain cells (including astrocytes, microglia, and radial glial cells), these cells are highly susceptible to ZIKV infection (Faizan et al., 2016; Laureti et al., 2018). Additionally, ZIKV can antagonize type I IFN signaling *via* up-regulating the expression of SOCS1 in a STAT1/STAT2-dependent manner (Chen et al., 2018) (**Figure 4B**). Several studies have shown that type I IFN signaling which is suppressed by various channels in ZIKV-infected cells plays an important role in antagonizing ZIKV infection (Grant et al., 2016; Kumar et al., 2016; Bowen et al., 2017). When the cells are stimulated by pathogenic invasion, the signaling of the defense system, such as Toll-like receptor (TLR) signaling pathways, leads to the outbreak of inflammatory cytokines. Cytokine signaling also drives up-regulation of AXL receptor expression, resulting in the induction of expression of suppressor of cytokine signaling (SOCS1 and SOCS3), thereby widely inhibiting the cascade of TLR and cytokine receptors (Lemke and Rothlin, 2008; Lemke, 2013).

In addition to the transmission of mosquito bites, sexual and vertical transmission may potentially increase the spread of ZIKV. In male mice, AXL is expressed in the testes and epididymides, but not in the prostate and seminal vesicles (Ma et al., 2016). Mouse model shows that ZIKV can cross the blood-testis barrier to replicate in the testes and epididymides and causes chronic inflammation and severe damage. In contrast, the prostate and seminal vesicles are protected from infection, which is consistent with the distribution of AXL (Ma et al., 2016). *In vitro* experiments also further confirm that AXL promotes ZIKV entry into human Sertoli cells (SC) and promotes its replication by antagonizing the interferon pathway (Kumar et al., 2018; Strange et al., 2019). But the results of AXL promoting ZIKV entry seems to be contradictory, which may be related to different host cells or different ZIKV strains (Ojha et al., 2019). During pregnancy, AXL varies substantially across donors, gestational age, and differentiation state, compared to another cofactor of ZIKV, T-cell immunoglobulin and mucin domain 1 (TIM-1), which is expressed consistently and uniformly (Tabata et al., 2016). In contrast, inhibition of AXL modestly reduces the infection of ZIKV, suggesting its secondary contribution in placental cells (Hamel et al., 2015; Fleming, 2016; Liu et al., 2016; Tabata et al., 2016). Because of differences in expression, the role of AXL during the ZIKV crossing the placental barrier is still uncertain. However, in human umbilical vein endothelial cells (HUVECs) with abundant AXL expression, the expression of other cofactors (DC-SIGN, L-SIGN, and TIM1) is poor, indicating that AXL is essential for ZIKV to infect HUVECs. Both ZIKV produced by insect cells and mammalian cells can use AXL to invade HUVECs and mediate productive infections. Nevertheless, only DENV or West Nile virus (WNV) produced by insect cells, not mammalian cells, can utilize AXL (Richard et al., 2017) (**Figure 4C**). This may explain why ZIKV can cross the placental barrier and cause vertical transmission while DENV and WNV do not have this ability. At present, it is still unclear as to why this phenomenon occurs. The possible reason is different lipid structure of mosquito cells and lower temperature used for virus production could affect virus assembly and expose more PS to bind Gas6. However, WNV virions produced at 37°C retain a closed smooth conformation. The flavivirus E protein structures exist as a dynamic and heterogeneous population. Greater membrane exposure could be achieved on ZIKV than on DENV or WNV if its structural proteins facilitate increased dynamic motion.

## Does ZIKV Really Need AXL

Although many studies have shown that AXL is a potential receptor for ZIKV infection, other studies have provided conflicting results. After *AXL^−/−^* mice are infected with ZIKV, high levels of ZIKV infection are still present in the testes and epididymis, which suggests that AXL is likely dispensable for ZIKV pathogenesis in the male reproductive tract (Govero et al., 2016). Hastings inoculated ZIKV on pregnant WT or *AXL^−/−^* mice and found no difference in ZIKV RNA levels in the brains and spleens of pregnant WT and *AXL^−/−^* mice. At the same time, there is no significant difference in the brain and placental tissue of fetal WT and *AXL^−/−^* mice (Hastings et al., 2017). These results prove that AXL is not essential for the vertical propagation of ZIKV. In a study of ZIKV tropic cells (NPCs), depletion of *AXL* fails to protect human neural progenitor cells and cerebral organoids from the infection of ZIKV (Wells et al., 2016). Other mouse models also show that pancreatitis, conjunctivitis, and eye infections caused by ZIKV infection are not related to AXL (Miner et al., 2016; Wang et al., 2017). To avoid errors caused by the differences between human AXL and murine AXL, Hela cells (AXL has been knocked out with CRISPR-Cas9 in advance) stably expressing murine AXL could restore the infection of ZIKV (Hastings et al., 2017). These findings call into a question about whether ZIKV really needs AXL in the process of infecting hosts. Another study suggests that AXL can cause microglial apoptosis in the brain of mice infected with ZIKV and mediate IL-1β expression, although it is not required for replication of ZIKV *in vivo* (Hastings et al., 2019). Although there are diametrically opposite results *in vivo* and *in vitro*, it cannot be easily denied that AXL is a candidate receptor for ZIKV. Instead, it illustrates the complex mechanism of AXL in the flavivirus replication process.

Combining these results, we speculate the mechanisms why *AXL^−/−^* mice can still be infected by ZIKV. a) Human AXL and murine AXL are different, although murine AXL can restore the sensitivity of human AXL-deficient cells to ZIKV. However, in mice, murine AXL has different effects on ZIKV infection, such as mediating the expression of interleukins and the apoptosis of nerve cells (Hastings et al., 2019). Interestingly, GBS caused by acute ZIKV infection may be associated with infection or autoimmune-mediated death of neuronal or glial cell. ZIKV-infected *Ifnar^−/−^* mice are characterized by acute and severe neuropathology and hindlimb paralysis. Both of them are likely related to AXL-mediated apoptosis of microglia (Li et al., 2016; Thiery et al., 2016; Krauer et al., 2017; Hastings et al., 2019). b) There are other receptors in the human cells hijacked by ZIKV. For instance, Tyro3, another protein of the TAM family, has also been shown to be one of the potential receptor for ZIKV and DENV (Meertens et al., 2012; Hamel et al., 2015; Onorati et al., 2016). Unilateral gene abolition of AXL cannot prevent the invasion of ZIKV due to receptors functional redundancy. While some other cells (such as HUVECs), due to the poor expression of other functional receptors, the abolition of AXL strongly inhibits ZIKV invasion (Brindley et al., 2011; Richard et al., 2017). c) There are other ZIKV receptor proteins expressed in mice but not in human cells. Because of the differences in species and the complexity of virus replication, the key target receptor for ZIKV infection in mice is not AXL but other molecules such as TIM1, DC-SIGN L-SIGN, or some unknown proteins. This requires multigene knockout mice and more work to verify this view in the future. d) As all mouse models not only lack AXL but also lack a key component of innate antiviral immunity (*Ifnar)*, loss of type I IFN signaling may mask the important role of AXL in ZIKV-infected mice. At present, *Ifnar^−/−^* mice are recognized as model mice for studying ZIKV because ZIKV preferentially infects cells with impaired abilities to produce type I IFN (Lazear et al., 2016; Hastings et al., 2017). In previous studies, ZIKV hijacked AXL to antagonize the type I interferon pathway to promote its infection, and the deficiency in *Ifnar* just weakened the need of ZIKV for AXL. e) The expression, distribution, and interaction of cell surface proteins in commercial immortal cells are usually different from those of primary cells *in vivo*, so it is not surprising that there are deviations between *in vivo* and *in vitro* experiments.

## AXL Receptor Blocking Drug Candidates Against DENV and ZIKV

Based on the worldwide population of ZIKV and DENV, there is an urgent need to develop effective interventions against them. AXL, as a common receptor candidate for ZIKV and DENV, is one of the potential targets for drug and inhibitor development. R428 is an anticancer drug known for targeting the kinase domain of AXL (Holland et al., 2010; Huey et al., 2016). *In vitro* model, it performs well in inhibiting ZIKV and DENV replication (Meertens et al., 2012; Meertens et al., 2017). Sarukhanyan improved the structure of R428 to form compound 1’ and 2’, which has a higher affinity with AXL than the original drug (Sarukhanyan et al., 2018). Warfarin is an anticoagulant drug that is intended to inhibit vitamin K epoxide reductase, and can also be used as an indirect blocker of AXL (Mega and Simon, 2015; Tormoen et al., 2020). The inhibition of epoxide reductase prevents carboxylation of glutamic acid residues in the epidermal growth factor domain of Gas6 responsible for attachment and fusion of enveloped viruses (Sarukhanyan et al., 2018). Through high-throughput genetic screens, it is found that BMS-907351 and BMS-777607 as AXL inhibitors could effectively inhibit the replication of ZIKV in human transformed osteosarcoma cells (Mega and Simon, 2015). Bhattacharyya also verified that BMS-777607 greatly inhibited activation of AXL and effectively reduced the replication of enveloped viruses in bone-marrow-derived DCs (Bhattacharyya et al., 2013). Besides, Pan found that nonsteroidal anti-inflammatory drugs such as aspirin, naproxen, ibuprofen, lornoxicam, and acetaminophen could inhibit ZIKV replication by degrading AXL *via* the ubiquitination pathway (Rausch et al., 2017; Pan et al., 2018) (**Figure 4D**). Although current inhibitors against AXL have been shown to inhibit the replication of ZIKV and DENV at the cellular level, whether these drugs can be used in clinical treatment is still unknown, and need more in-depth research.

## Other Remaining Unresolved Issues

With the outbreak of ZIKV, the interactions of AXL and flaviviruses are an active area of investigation, but there are still many doubts. a) How flaviviruses may incorporate PS in their membrane? It is known that the lumenal leaflet of ER membrane contains PS, which suggests PS can be incorporated into the surface of the virus particle when the virus is replicating (Leventis and Grinstein, 2010; Perera-Lecoin et al., 2013). Viruses can also induce apoptosis in various ways, subsequently expose PS on virus-producing cells and budding from the membrane of apoptotic cells (Nour et al., 2013; Chua et al., 2019). But It is worth noting that the presence of PS alone is not enough for enveloped viruses to enter by apoptotic mimicry. Because influenza A virus (IAV) and coronaviruses, which also have PS, are not enhanced by AXL (Jemielity et al., 2013; Moller-Tank and Maury, 2014). b) How Gas6 accesses virion-associated PS? As described before, why DENV or WNV produced by insect cells can use AXL, but not mammalian cells? It is generally believed that the virus structure is static, but the E protein structures exist as a dynamic and heterogeneous population, which contributes to the atomic model of virions that breath over time (Kuhn et al., 2015; Barrows et al., 2018). Viral breathing can be affected by the environment or viral genetic factors (Lewis et al., 1998; Austin et al., 2012; Kostyuchenko et al., 2014; Dowd et al., 2015; Lim et al., 2017; Barrows et al., 2018). At 28°C, mosquito cells produce smooth herringbone conformation as the dominant mature form of DENV viral particles. At 37°C, DENV particles appear unstable bumpy structure with broken, deformed, of different sizes, or in aggregates (Zhang et al., 2013b). The change of the conformation may expose patches of the virion membrane, making it easier to bind Gas6. In contrast, WNV particles maintain a smooth closed conformation at 37°C (Mukhopadhyay et al., 2003). The conformational change of viral particles caused by temperature does not seem to explain this phenomenon in all flaviviruses. Another phosphatidylserine receptor TIM-1 can directly interact with PS of DENV and WNV to promote infection (Meertens et al., 2012; Oliveira and Peron, 2019). Compared with TIM-1, Gas6 is a bigger protein, it may require larger exposed patches of the viral membrane, which may be more available for ZIKV than for DENV or WNV (Kostyuchenko et al., 2016; Richard et al., 2017; Hasan et al., 2018). c) Is AXL a common receptor for all flaviviruses, and does AXL perform other functions during flavivirus-infection? Previous research has indicated that AXL protects against WNV infection by maintaining blood-brain barrier (BBB) integrity, suggesting the new role of AXL in flavivirus research (Miner et al., 2015). AXL not only promotes the entry of EBOV but also suppresses its release in the later stages of replication (Shimojima et al., 2006; Li et al., 2014).

## Conclusion

In this review, we summarize the biological characteristics of DENV, ZIKV, and AXL, and the biological functions of AXL in the invasion of cells by DENV and ZIKV. At present, it is believed that AXL mainly plays a role in two ways in the process of virus replication, a) promoting virus binding and internalization and b) promoting virus replication by activating AXL to antagonize the type I interferon pathway. But the contradictory results shown in the mouse experiments seem to close the door for the study of AXL and flaviviruses. Future research needs to focus on discovering the causes of the differential results *in vitro* and *in vivo*, and the mechanism by which flaviviruses expose PS to bind phosphatidylserine receptors. Through an in-depth understanding of the above issues, the development of AXL inhibitors that can be used in clinical treatment may be a new antiviral strategy.

## Author Contributions

RC designed this work and revised the manuscript. SX wrote the manuscript, SX and HZ revised the manuscript within Interactive Review, ZL and XY provided critical suggestions of the manuscript. All authors contributed to the article and approved the submitted version.

## Funding

This work is funded by the National Key Research and Development Plan of China (2016YFD0500402) and SRT Program for National Undergraduates of Nanjing Agricultural University (201910307019Z).

## Conflict of Interest

The authors declare that the research was conducted in the absence of any commercial or financial relationships that could be construed as a potential conflict of interest.
